# Resilience assessment of Puerto Rico’s coral reefs to inform reef management

**DOI:** 10.1371/journal.pone.0224360

**Published:** 2019-11-05

**Authors:** David A. Gibbs, Jordan M. West

**Affiliations:** 1 Oak Ridge Institute for Science Education fellow at U.S. Environmental Protection Agency, Washington, D.C., United States of America; 2 U.S. Environmental Protection Agency, Office of Research and Development, Washington, D.C., United States of America; University of Plymouth, UNITED KINGDOM

## Abstract

Globally increasing sea surface temperatures threaten coral reefs, both directly and through interactions with local stressors. More resilient reefs have a higher likelihood of returning to a coral-dominated state following a disturbance, such as a mass bleaching event. To advance practical approaches to reef resilience assessments and aid resilience-based management of coral reefs, we conducted a resilience assessment for Puerto Rico’s coral reefs, modified from methods used in other U.S. jurisdictions. We calculated relative resilience scores for 103 sites from an existing commonwealth-wide survey using eight resilience indicators—such as coral diversity, macroalgae percent cover, and herbivorous fish biomass—and assessed which indicators most drove resilience. We found that sites of very different relative resilience were generally highly spatially intermixed, underscoring the importance and necessity of decision making and management at fine scales. In combination with information on levels of two localized stressors (fishing pressure and pollution exposure), we used the resilience indicators to assess which of seven potential management actions could be used at each site to maintain or improve resilience. Fishery management was the management action that applied to the most sites. Furthermore, we combined sites’ resilience scores with projected ocean warming to assign sites to vulnerability categories. Island-wide or community-level managers can use the actions and vulnerability information as a starting point for resilience-based management of their reefs. This assessment differs from many previous ones because we tested how much information could be yielded by a “desktop” assessment using freely-available, existing data rather than from a customized, resilience-focused field survey. The available data still permitted analyses comparable to previous assessments, demonstrating that desktop resilience assessments can substitute for assessments with field components under some circumstances.

## Introduction

The widely varying responses of coral reefs to stressors such as increased sea surface temperatures (SST) may be due to many factors, such as variation in a reef’s exposure to the stressor, sensitivity to the stressor, and capacity to adapt [1]. While the attributes of sensitivity and adaptive capacity are considered defining components of resilience [2], resilience can be defined as a system’s capacity to absorb recurring disturbances (resistance) and regain essentially the same state and processes as before (recovery) [3]. A reef’s resilience can be characterized by the trajectory it takes following disturbance; high resilience reefs will tend towards dominance by corals, while low resilience reefs will tend towards dominance by other organisms [3]. Although resilience is relevant with respect to any kind of environmental disturbance, for coral reefs a major concern is resilience to rising SST and its interaction with local stressors [4]. Prolonged elevated SST can disturb reefs by causing corals to bleach (lose the symbiotic dinoflagellates that provide a large share of their nutrients), potentially leading to coral mortality.

Resilience-based management has become an important principle for managing reefs with respect to multiple stressors at multiple spatial scales. However, to manage reefs based on resilience, managers must know which reefs or areas tend to be more resilient and which tend to be less [3]. The resilience of reefs can be identified by conducting a resilience assessment, the goal of which is to inform reef management at one or more spatial scales [5]. Resilience is generally defined relative to a pool of sites or an area of interest, rather than in absolute terms. There are two main reasons to conduct resilience assessments: 1) to help target where to engage in various environmental management actions, and 2) to evaluate the effectiveness of reef management and conservation actions taken to increase resilience [6]. Potential outputs of resilience assessments include the spatial distribution of sites of varying resilience, the range in relative resilience, drivers of resilience [7], and management actions that can be informed by resilience [8]. These can be highly localized actions, like selecting sites where outplanting corals will be most beneficial and likely to succeed, or broader-scale actions, like designing marine protected areas to include sites with a range of resilience [9].

Resilience assessments are based on identification of measurable properties (i.e., indicators) of coral reefs that relate directly or indirectly to how reefs respond to disturbance [7], or to the probability that reef condition will change following disturbance [3]. Resilience indicators can be used to help identify which properties of reefs drive high resilience in an area, which is important for understanding how reefs respond to the stressor of interest. Ideally, resilience indicators capture reef state (pattern) and function (process), both of which contribute to sensitivity and adaptive capacity [6]. It should then follow that management actions that increase these indicators (such as the creation of herbivore management areas to increase herbivore biomass [10]) will increase reef resilience.

Methods for reef resilience assessments were formalized by [6]. Their detailed methods included datasheets with over 55 potential indicators and numeric/qualitative criteria for five different resilience levels for each indicator. [11] used 19 indicators in a small part of the southern Great Barrier Reef; each indicator could be assigned one of three weights based on a review of the literature and one of four resilience categories for each site. [12] used literature reviews and expert opinion to identify eleven of the most important resilience indicators, then scored those indicators on a Likert scale for a resilience assessment in Java, Indonesia. [7] described a simplified assessment method that has since been adapted for St. Croix, U.S. Virgin Islands [13], the Commonwealth of the Northern Mariana Islands [8,9], West Hawai’i, USA [14], and the Florida reef tract [15] in an effort to conduct resilience assessments for all U.S. coral reef jurisdictions using similar methods. In addition to mapping resilience by survey site, assessments have generally included management recommendations, such as where coral restoration is most likely to be effective and where protected areas should be enforced or established.

We assessed the resilience of coral reefs to ocean warming around the U.S. Caribbean commonwealth of Puerto Rico using the methods of [7] and [2]. The assessment encompasses the entire island, as well as the outlying islands. We adapted their basic methods to suit local context and expanded upon other jurisdictions’ assessments. Unlike some previous resilience assessments, this assessment is not based on a field survey designed specifically to measure resilience because we wanted to explore the feasibility of the approach using only readily available data. We therefore limited ourselves to using existing commonwealth-wide data. This gave us the opportunity to explore what level of understanding of a jurisdiction’s resilience could be achieved through a desktop-only assessment instead of through a custom-survey assessment. Because field surveys tailored to resilience assessments are not feasible in all situations, this serves as a test of what can be done using desktop methods alone. Additionally, this contributes to resilience-based management of Puerto Rico’s coral reefs by the transparent characterization of resilience and potential reef management actions.

## Methods

### Indicator data acquisition

We calculated resilience indicators from the National Coral Reef Monitoring Program (NCRMP) survey of Puerto Rico’s coral reefs during summer 2014, the most recent comprehensive survey of Puerto Rico’s reefs [16]. This survey used a probabilistic design in which sampling location was stratified by depth, habitat, region of Puerto Rico, and presence of a marine protected area. As a probabilistic survey, the survey was designed to represent Puerto Rico’s coastal waters as a whole rather than to include the full range of reef quality, long-term monitoring sites, or particular reefs of interest to resource managers, the tourism sector, etc. The survey included 230 sites at which were conducted 25x4 m fish belt transects [17]; topographic complexity surveys using 24 vertical relief measurements in the same transects [18]; and 20 m line-point intercept (LPI) benthic surveys [19]. In addition, 10x1 m coral colony demographic surveys were conducted at 111 of the sites using the same stratification system [20]; these recorded species, dimensions, and health status for all colonies greater than 4 cm diameter.

### Indicator and site selection

Indicators for resilience to increasing SST may not be the same as indicators of resilience to other large-scale climate stressors, such as ocean acidification (OA) or changing storm patterns. For example, the dozens of indicators of [12] and [6] focused on resilience to warmer oceans, rather than on other consequences of planet-scale changes. We intended our indicators to do likewise. Furthermore, we wanted to select indicators that best represent Puerto Rico’s reefs in particular, as recommended in previous assessments [6–8,21]. We first narrowed the universe of potential resilience indicators by comparing those used in assessments in other locations with reef biocriteria metrics for Puerto Rico that were developed for a biological condition gradient (BCG) project [22] (S1 Table). The BCG is an approach for assessing the condition of an ecosystem on an absolute scale using a suite of indicators selected by experts. A BCG is under development for Puerto Rico’s coral reefs [22] and was relevant for selecting indicators because it is another indicator-based approach to measuring reef condition. We determined which previously used resilience indicators and which Puerto Rico biocriteria metrics we could calculate from the available data. Because NCRMP was not designed for a resilience assessment, not all the data included in other resilience assessments were available for Puerto Rico, primarily coral recruitment.

We then conducted a series of webinars with five experts on Puerto Rico’s coral reefs, during which we asked for recommendations on what indicators to use (following methods similar to those of [6] and [13]). The experts recommended that all of the indicators used in previous assessments except for one should be included in the Puerto Rico assessment. The recommended indicators were: Simpson diversity of scleractinian corals, fraction of scleractinian colonies without disease, percent cover of scleractinian corals, percent cover of macroalgae, total herbivorous fish biomass, and the average thermal tolerance of scleractinian corals. SST variation was deemed unlikely to be an important resilience indicator in Puerto Rico because of the relatively consistent variation across the study area, making it the only indicator with available data used in previous assessments to not be recommended as an indicator for this assessment. For consistency with previous assessments and because of evidence for the role of temperature variability in bleaching response (e.g., [23]), we calculated resilience with and without the temperature variation indicator. The experts agreed that coral recruitment would be valuable as a resilience indicator but noted that it could not be calculated from the available data because NCRMP protocols did not record colonies <4 cm maximum diameter. The panel also recommended that rugosity be included as an indicator. This list of indicators is more ecologically focused than some other resilience assessments that used different methods (e.g., [4]), but it is in line with other assessments based on [7].

Because Simpson diversity, fraction of corals without disease, and average coral thermal tolerance required colony-specific information from the demographic survey, we used only survey sites at which a demographic survey was conducted. Of the 111 such sites, NCRMP surveyed eight sites at which there were no colonies large enough to collect demographic data (>4 cm diameter). This left 103 sites with adequate data for calculation of all indicators. About half of these sites were around Puerto Rico’s main island and half were around smaller islands, like Vieques, Culebra, and Mona (mean maximum depth of all sites: 13 m, standard deviation: 6.4 m).

### Indicator and resilience score calculation

We followed the methods of [7] and [2] to calculate resilience indicators. For all indicators, larger values mean greater resilience. A brief description of the calculation of each indicator is below:

- *Coral diversity index*: The Gini-Simpson diversity index (1 –Simpson index) [24] of all scleractinian colonies >4 cm maximum diameter.
- *Fraction of hard coral colonies without disease*: The number of colonies >4 cm diameter without any disease divided by the total number of colonies >4 cm diameter.
- *Scleractinian coral percent cover*: The percent of the points in the LPI survey at which scleractinian corals were found.
- *Macroalgae cover*: The proportion of the points in the LPI survey at which macroalgae were found. NCRMP categories for this indicator included: *Dictyota*, *Lobophora*, *Peysonnellia*, *Halimeda*, MacroOtherCalcareous (i.e., upright calcareous algae, not encrusting algae), and MacroOtherFleshy. We subtracted this value from 1 so that larger values meant less macroalgae.
- *Rugosity*: Twenty-four vertical relief measurements were taken within each 25x4 m transect, grouped into six categories (<20 cm, 20–50 cm, 50–100 cm, 100–150 cm, 150–200 cm, and >200 cm), and averaged for each site to create a single rugosity measurement.
- *Herbivorous fish biomass*: NCRMP fish surveys recorded fish abundances in 5 cm size classes (e.g., 10–15 cm, 15–20 cm). We classified the following species as herbivores: *Acanthurus bahianus*, *A*. *chirurgus*, *A*. *coeruleus*, *Kyphosus sectatrix*, *Scarus iseri*, *Sc*. *taeniopterus*, *Sc*. *vetula*, *Sparisoma atomarium*, *Sp*. *aerofrenatum*, *Sp*. *chrysopterum*, *Sp*. *rubripinne*, and *Sp*. *viride*. We used average length-weight coefficients (*a* and *b*) for each species, which were retrieved from fishbase.org (downloaded June 27, 2016) in the fish length-mass equation w = a*L^b^, where w = weight and L = fish length. We used the midpoint of the length class for that fish’s length (e.g., 12.5 cm for the 10–15 cm class), except for fish that were <5 cm, which we assigned a length of 3 cm. We summed the biomass of all herbivorous fish at each site for this indicator.
- *Average thermal tolerance of scleractinian corals*: We used the Puerto Rico BCG assignments of taxa to five thermal tolerance categories for this indicator [22]. A few rare species found at a few sites did not have thermal tolerances assigned in the BCG report. For these taxa, we used the averaged values of their congeners. We applied these tolerance values to all colonies in the demographic surveys to calculate the average tolerance of colonies >4 cm diameter at each site.
- *Sea surface temperature variation*: We used detrended standard deviation in SST over 1985–2012, using degree-heating week (DHW) data from NOAA Coral Reef Watch, from [25]. The resulting grid was at 1/24-degree resolution (about 20 km^2^). We assigned each survey station the SST variation value of the pixel it was in; the nineteen stations not within a pixel because they were too close to the coast for the model were assigned the value of the pixel to which they were closest (S. Heron, pers. comm.). Due to the variation in sites’ depths, different sites will actually experience the reported variation differently.

After we calculated the raw indicator values, we rescaled each indicator to 1, with 1 being the highest value of that indicator found at any of the 103 sites. Finally, we averaged the rescaled indicators (with and without the temperature variation indicator) to obtain a composite relative resilience score for each site. We rescaled those values to 1, so that the site with the highest resilience among those surveyed had a value of 1. Rescaling individual indicators and the resilience score to 1 emphasizes that they are relative to the surveyed sites. Unless otherwise specified, further analyses and results reported below are without the temperature variation indicator, per the experts’ recommendation.

We ranked the composite resilience scores between 1 and 103 and divided them into quartiles. Our use of quartiles differs from some previous resilience assessments [8,14,15], which categorized sites based on the average and standard deviation of the resilience scores or divided sites into resilience and stressor categories based on fixed numeric cutoffs [13]. We used quartiles to assign sites to resilience categories because of the skewed distribution of resilience scores. Our use of quartile-based resilience categories instead of resilience scores or standard deviation-based resilience categories further emphasizes the relative nature of the resilience assessment. This emphasis on relativity is similar to that of [26], who identified oases of healthy reefs within degraded areas using distributions of surveyed sites.

We examined the spatial distribution of resilience scores in two ways. First, we used the Spatial Autocorrelation (Global Moran’s I) geoprocessing tool in ArcGIS Pro v2.0 to assess whether sites were aggregated, randomly arranged, or over-dispersed by resilience quartile. We did this using both inverse distance and inverse distance squared for the spatial relationship conceptualization (the rate at which sites near each other are expected to be similar) because neither one is obviously more conceptually applicable, and with an infinite distance threshold (i.e., all sites were compared to all other sites), again because there was no empirical basis for limiting comparisons to a specific distance. Second, we used the inverse distance weighting raster interpolation tool in ArcGIS Pro v2.0 to interpolate resilience scores for pixels 0.02 x 0.02 degrees (approximately 2.2 x 2.2 km) within approximately 10 km of the coast (see S1 Text for tool parameterization details). This create a resilience surface using the resilience scores of nearby sites. Because NCRMP survey sites are chosen probabilistically, they fit the assumptions of this tool. Note that this analysis interpolates resilience scores for areas with non-reef habitat, like soft bottom habitat.

### Relationships between individual indicators and their contributions to resilience

Looking only at composite resilience scores masks the variation behind those scores, as well as important information from the indicators themselves. Indeed, knowing the relative influence of different indicators on resilience scores can help with designing monitoring programs and management actions [8]. Therefore, we examined the contribution of individual indicators to the composite resilience scores in three ways. First, we created box plots of rescaled indicators to visualize how much each indicator varied. The more variable an indicator is, the more useful it is for distinguishing resilience between sites [2]. Second, we created a matrix of Spearman rank-correlation coefficients between all indicators. We used Spearman correlation coefficients because the distributions of several of the indicators were heavily skewed. Third, we performed an exploratory factor analysis (EFA) with a varimax rotation on all the indicators to identify latent variables in the resilience assessment. We retained factors with an eigenvalue > 1 using a scree plot for latent variable interpretation. All calculations and statistical analyses were performed in R [27].

### Indicator weighting sensitivity analysis

In the above calculation of resilience scores, all indicators were equally weighted. In other words, we assumed they contribute equally to reef resilience. Because this is probably an unrealistic assumption, but the actual importance of each indicator is unknown [7,11], we assessed to what extent the weighting of indicators affected resilience rankings and quartiles. In addition to the unweighted resilience scores, we calculated resilience scores using nine different indicator weighting systems (Table 1). This is not an exhaustive list of weighting options, nor do we think that any one of them is correct, just as we do not propose that all indicators equally measure reef resilience. Rather, the weighting systems are supposed to show to what extent the resilience scores are robust to the null model that each indicator equally represents reef resilience. To calculate the weighted resilience scores, we multiplied each indicator by its weight and averaged those weighted values for each site, then rescaled to 1 (as with the unweighted indicators).

**Table 1:** Weighting systems for resilience indicators. A larger value means the indicator is given more weight in calculation of the resilience score. Weighting systems 3–9 are based on Table 2 in [12]. Root mean square errors (RMSE) are from the comparison of weighted indicator resilience ranks against unweighted indicator resilience ranks.

Weighting system	Weighting description	Simpson diversity index	Fraction not diseased colonies	Percent hard coral cover	Percent not macroalgae cover	Rug-osity	Total herbivore biomass	Temp-erature variation	Average coral thermal tolerance	Root mean square error (RMSE)
**0**	Unweighted	1	1	1	1	1	1	1	1	N/A
**1**	Random- up to 2x weighting	1.16	1.65	1.94	1.34	1.03	1.43	1.39	1.2	4.19
**2**	Random- up to 4x weighting	1.02	2.35	1.48	3.91	2.81	3.11	1.55	2.72	11.9
**3**	"Resilience- perceived importance" ranked, integer increments (1–8)	6	4	2	3	1	5	7	8	14.2
**4**	"Resilience- perceived importance" ranked, 0.1 increments (1–1.7)	1.5	1.3	1.1	1.2	1	1.4	1.6	1.7	3.16
**5**	"Resilience- perceived importance" ranked, 0.2 increments (1–2.4)	2	1.6	1.2	1.4	1	1.8	2.2	2.4	4.98
**6**	"Resilience- perceived importance" ranked backwards, 0.2 increments (2.4–1)	1.4	1.8	2.2	2	2.4	1.6	1.2	1	3.09
**7**	Relative to lowest average of “Resilience”, “Resistance”, and “Recovery” in “Perceived importance” and “Scientific evidence” for the indicators used in this assessment	1.38	1.28	1.17	1.40	1.00	1.41	1.65	1.84	4.37
**8**	Relative to lowest average of average of “Resilience”, “Resistance”, and “Recovery” in “Perceived importance”, “Scientific evidence”, and “Feasibility” for the indicators used in this assessment	1.31	1.21	1.25	1.39	1.00	1.34	1.53	1.68	3.66
**9**	Relative to lowest average of "Resilience- perceived importance" and "Resilience- scientific evidence" for the indicators used in this assessment	1.44	1.34	1.10	1.41	1.00	1.46	1.76	1.98	4.85

Weighting systems 1 and 2 randomly assigned weights within specified ranges to indicators while systems 3–9 were based on Table 2 in [12], which provides relative importance values for reef resilience indicators based on a review of literature and expert opinion. The weights of indicators in systems 3–9 are ordered based on their order in the “Resilience- perceived importance” column in that table. For example, in our weighting system 3—our most extreme system—the highest-ranked indicator from Table 2 of [12] (coral thermal tolerance) is given eight times as much weight in the resilience score calculation as is the lowest ranked indicator used from that table (rugosity/topographic complexity). Systems 4 through 6 are essentially less extreme versions of system 3. Systems 7 through 9 rescale the indicators to the lowest-ranked of the indicators used in this assessment (rugosity/topographic complexity) according to values from Table 2 in [12]. Thus, systems 7 through 9 are the most ecologically grounded of all the scaling systems.

After we applied the weighting systems, we calculated by how much the resilience rank of each site changed under each weighting system and how many sites changed their resilience quartile. We used these to assess how much the various weighting systems affected resilience scores.

### Stressor calculation

Increasing SST interacts with and compounds the effects of localized stressors [28]. Previous resilience assessments, such as [29] and [9], have recognized these localized stressors as important moderators of resilience. Thus, in addition to the above resilience indicators, we also calculated two stressors that can be used to inform reef management: potential fishing pressure, relative land-based pollution loads (a combination of nitrogen and sediment loads).

*Potential fishing pressure*: [30] created a grid of estimated potential line, net, trap, and dive fishing pressure around Puerto Rico by surveying fishermen (see also [31]). We assigned the total fishing pressure of each grid cell to the survey stations. Nine stations around Vieques and Culebra islands were so close to shore that they were not in the fishing grid; we assigned those stations the total fishing pressure of the nearest grid cell.

*Relative land-based nitrogen and sediment levels*: We estimated relative land-based pollutant loads at survey sites in two steps: 1) loads at the mouths of Puerto Rico’s rivers and streams, and 2) dispersal of those pollutants to survey sites. We used the free and open watershed modeling software OpenNSPECT to calculate loads at river mouths [32], following the data acquisition and model manuals [33]. Although OpenNSPECT produced numerical values, its outputs are better interpreted relative to each other rather than as actual estimates of pollutants. With this in mind, we validated OpenNSPECT’s watershed flow and sediment outputs against US Geological Survey (USGS) stream gage data. Next, we used a simple LBSP dispersal model based on the distance between reef survey sites and the seven nearest river mouths modeled by OpenNSPECT to release more than 183 kg N per year (0.5 kg N per day) to estimate relative loads at NCRMP sites. To obtain a single land-based relative pollution estimate for each site, we averaged the nitrogen and sediment estimates and rescaled to one [9], essentially producing relative LBSP scores for each site. This resulted in 59 sites around the main island because our data sources were restricted to the main island. For additional information, refer to S2 Text.

### Management option queries

We used a subset of indicators, resilience scores, and stressors at each survey site to identify potential management actions that could be used to improve or maintain reef resilience through a decision support framework (Table 2) (akin to [8]). The queries are meant to be preliminary; sites would need further investigation and a detailed implementation plan before any of the management actions could be instituted. Moreover, the thresholds or criteria used in the queries are flexible; they can be changed based on needs and context. These queries simply provide an idea of where certain kinds of actions might be more useful and where combinations of actions might be applied. The actions in Table 2 aim to restore, protect and/or improve reef resilience by: 1) restoring reef species, structures and functions where they have been degraded; 2) protecting reefs; and 3) improving recovery from bleaching and disease events. Customized queries can be made using the data in S2 Table and the provided R script (S1 Information File).

**Table 2:** Management action queries based on resilience indicators, resilience scores, and stressors. Not all queries use all three types of information.

Query name (abbreviation)	Query criteria
Coral restoration (R)	1) Hard coral cover is lower than the average, and 2) site resilience is in the upper two quartiles
Land-based pollution management (L)	1) Land-based sources of pollution are higher than the average, and 2) site resilience is in the upper two quartiles
Tourism outreach (T)	1) Coral diversity is higher than average, 2) algae cover is lower than the average, and 3) herbivore biomass is higher than the average
Fishery management (F)	1) Herbivore biomass is lower than the average, and 2) fishing pressure is higher than the average
Reef protection (P)	1) LBSP is moderate, 2) fishing is moderate, and 3) resilience is in the upper two quartiles
Bleaching management (B)	1) Bleaching resistance is lower than the average, and 2) herbivore biomass is lower than the average
Disease management (D)	1) Coral cover is higher than average, and 2) disease prevalence is higher than the average

### Bleaching exposure and vulnerability

We overlaid the resilience results on downscaled estimates of the year by which annual significant bleaching (ASB) will occur under the climate change representative concentration pathway (RCP) 8.5 radiative forcing scenario from [34]. Combining resilience (sensitivity plus adaptive capacity) and temperature increase (exposure) allowed us to approximate reef vulnerability [2]. Because of the relatively narrow range of onset years for ASB (10 years), we divided sites into two exposure categories: 2036–2041 (60 sites) and 2042–2046 (43 sites). Twenty-seven stations did not fall within the ASB onset projection raster; we assigned those sites to the nearest raster cell. We classified sites with high ASB exposure and low resilience as having high vulnerability, sites with low ASB exposure and high resilience as having low vulnerability, and sites with both high ASB exposure and resilience or both low ASB exposure and resilience as having moderate vulnerability. As in [2], a step up in exposure (from low to high) is assumed to be equivalent to a step down in resilience (from high to low) in terms of vulnerability. As with resilience and the stressor estimates, vulnerability is relative among the sites surveyed.

## Results

### Indicators and resilience scores

**Fig 1:**
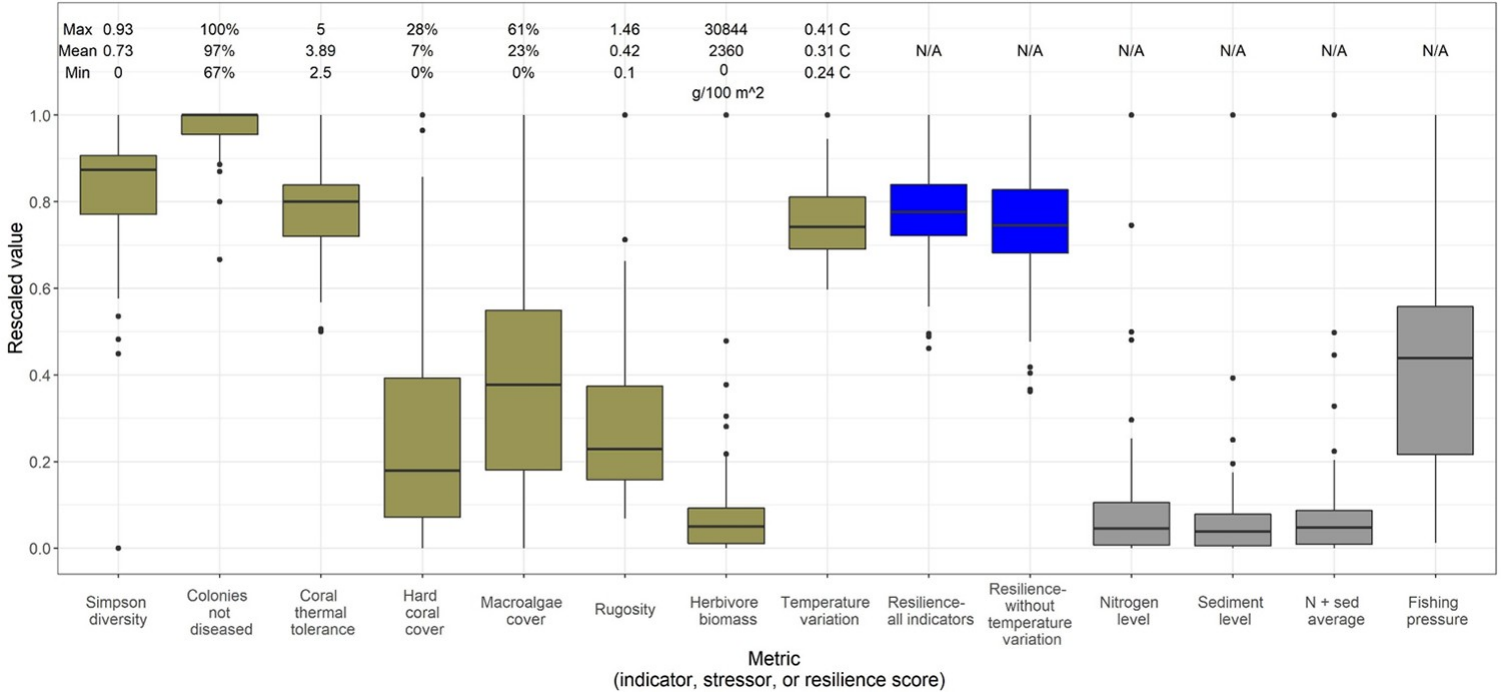
Rescaled indicators, unweighted resilience scores, and stressors for 103 reef survey sites around Puerto Rico. Indicators have the raw minimum and maximum values found at all 103 sites above their boxplots; numbers without units are unitless indicators. Resilience scores and stressors do not have raw minimum and maximum values because they are calculated relative to the maximum at the surveyed sites. Brown bars are resilience indicators, blue bars are resilience scores, and grays bars are stressors.

The distribution of indicators, stressors and unweighted resilience scores are shown in Fig 1. Following the experts’ suggestions of which indicators to use, we focused on resilience scores without the temperature variation indicator, but we also calculated resilience scores with the temperature variation indicator for comparability with previous assessments that used that indicator [8,9,13,15]. There was slightly greater variation in resilience scores without the temperature indicator (standard deviation = 0.127) than with it (standard deviation = 0.108) but a very strong correlation between the two resilience scores (Pearson’s r = 0.99). Unless otherwise specified, further results are without the temperature variation indicator.

**Fig 2:**
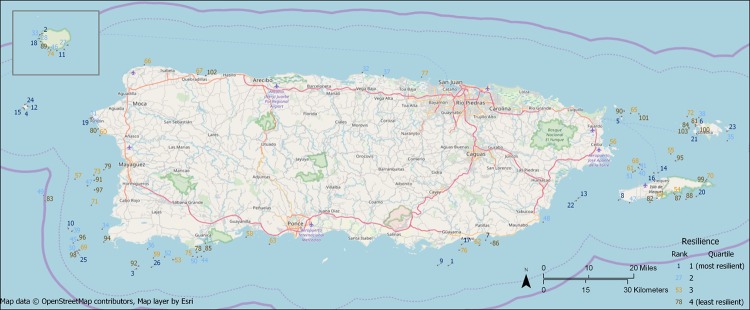
Survey sites (n = 103) with resilience rank scores, calculated without using the temperature variation indicator. Isla de Mona is the inset in the top left. Resilience quartiles are based on resilience scores without the temperature variation indicator.

**Fig 3:**
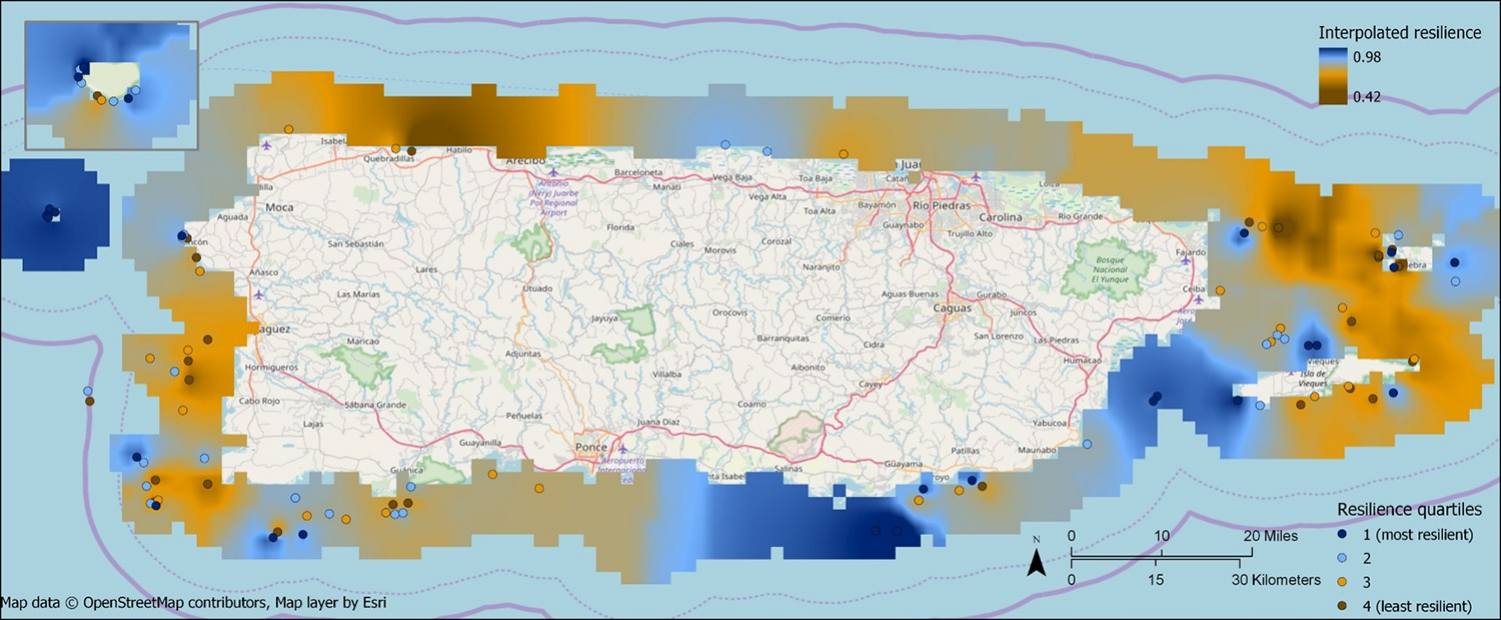
Resilience scores interpolated up to 10 km from the coast using inverse distance weighting of the resilience scores of survey sites. Sites outside the interpolation raster were not used in the interpolation. Not all of the interpolated locations historically had coral reefs or do currently have them.

Survey sites were randomly distributed across the survey area in terms of resilience quartile using both the inverse distance and inverse distance squared methods, although there was a non-significant tendency towards clustering (inverse distance: Moran’s Index = 0.0650, Expected Index = -.009804, z-score = 1.503, p-value = 0.13; inverse distance squared: Moran’s Index = 0.315, Expected Index = -.009804, z-score = 1.672, p-value = 0.09). Some areas did appear to tend towards higher or lower resilience (Fig 2). For example, all four of the sites in Desecheo National Wildlife Refuge to the west of Puerto Rico were in the highest resilience quartile, while five of the six sites near Vieques National Wildlife Refuge to the east of Puerto Rico were in the lowest two quartiles and six of the seven sites around Cabo Rojo/Mayaguez on the west coast of Puerto Rico were in the lowest two quartiles. On the other extreme, the four southwestern-most sites, all within 1500 m of each other, span the four quartiles, ranging from resilience ranks 25 to 98. To the extent there is clustering of sites by resilience, it can be seen in Fig 3, in which interpolated resilience is higher in some patches than in others.

### Relationships between individual indicators and their contributions to resilience

Many of the coral-related indicators were moderately positively or negatively correlated with each other (Table 3). Interestingly, coral thermal tolerance and Simpson diversity had the strongest negative correlation (rho = -0.54). Herbivore biomass was most strongly correlated with two coral measures (rugosity and hard coral cover), while temperature variation did not have any correlations >|0.19|. Macroalgae cover did not have any correlations >|0.17|.

**Table 3:** Spearman rank correlation coefficients between resilience indicators at 103 survey sites.

	**Simpson diversity index**	**Fraction not diseased colonies**	**Percent hard coral cover**	**Percent not macroalgae cover**	**Rugosity**	**Total herbivore biomass**	**Temperature standard deviation**	**Average coral thermal tolerance**
**Simpson diversity index**	1.00	-0.11	0.31	0.08	0.27	0.16	-0.11	-0.54
**Fraction not diseased colonies**		1.00	-0.28	-0.02	-0.05	-0.13	0.01	-0.02
**Percent hard coral cover**			1.00	0.14	0.58	0.33	-0.17	-0.24
**Macroalgae cover**				1.00	0.17	-0.01	-0.07	-0.09
**Rugosity**					1.00	0.59	-0.11	-0.37
**Total herbivore biomass**						1.00	-0.19	-0.14
**Temperature variation**							1.00	-0.03
**Average coral thermal tolerance**								1.00

In terms of variation within each indicator, diseased colonies and coral thermal tolerance contributed the least to distinguishing between the resilience of sites due to their relatively small amount of variation (Fig 1). Four indicators’ rescaled ranges included the smallest possible value (0), meaning that their rescaled values extended between 0 and 1 (Simpson diversity, hard coral percent cover, macroalgae percent cover, and herbivore biomass). Those indicators would be expected to be most useful in distinguishing reef resilience.

The exploratory factor analysis identified four factors with eigenvalues > 1 (χ^2^ = 1.4, df = 2) (Table 4). The first factor explained 17.4% of the variance, the second factor explained 14.8%, the third factor explained 11.6%, and the fourth explained 7.5%. The first factor was dominated by hard coral cover and the other three indicators with loadings >|0.1| were all coral-related, so it could therefore be interpreted as a coral-driven factor. The second factor was dominated by herbivore biomass but the other three indicators with loadings >|0.1| were also coral-related, so the second factor could be interpreted as herbivores and their habitat requirements. The third factor was dominated by average coral thermal tolerance, with temperature variation and two coral indicators also registering >|0.1|. This might be interpreted as the aggregate temperature sensitivity of the coral community. Finally, the fourth factor is not dominated by any single indicator. It is also the only factor where the loading of macroalgae percent cover loading is >|0.1|. This factor might be a catch-all for benthic community structure.

**Table 4:** Loadings from an exploratory factor analysis using varimax rotation. Four factors had eigenvalues > 1 and were therefore extracted. Blank cells had loadings <|0.1|.

	Factor 1	Factor 2	Factor 3	Factor 4
**Simpson diversity index**	0.366	0.114	-0.211	0.200
**Fraction not diseased colonies**				0.174
**Percent hard coral cover**	0.982		0.153	
**Percent not macroalgae cover**				0.506
**Rugosity**	0.393	0.384		0.489
**Total herbivore biomass**		0.995		
**Temperature variation**			-0.177	
**Average coral thermal tolerance**	-0.343	-0.176	0.905	-0.167
**Sum of squares loadings**	1.393	1.188	0.931	0.600
**Proportion variance explained**	0.174	0.148	0.116	0.075

### Indicator weighting sensitivity analysis

**Fig 4:**
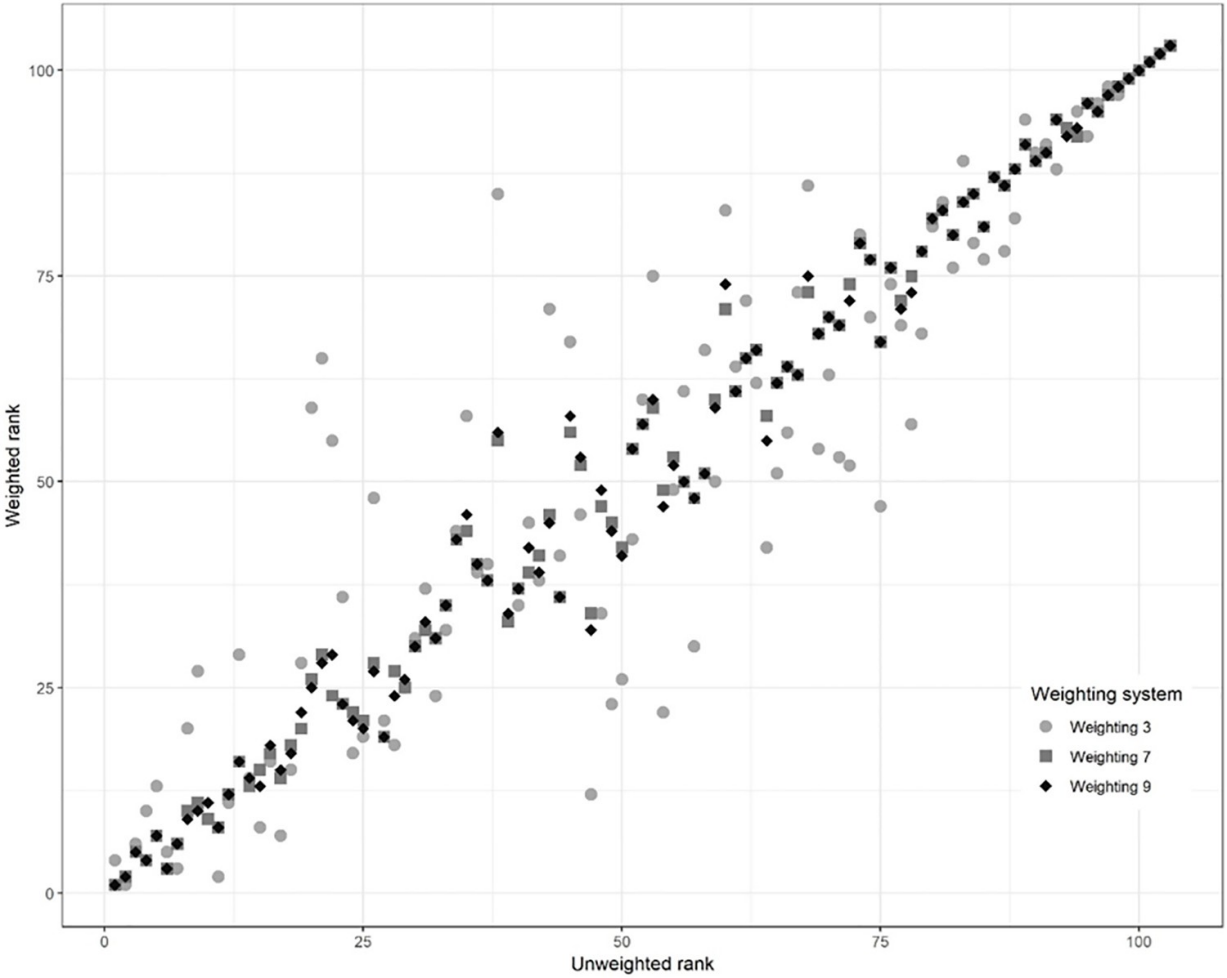
Relationship between unweighted resilience ranks and select weighted resilience ranks for 103 sites around Puerto Rico. For descriptions of each weighting system, refer to Table 1. Root mean square error (RMSE): Weighting 3–14.2, Weighting 7–4.37, Weighting 9–4.85.

For all weighting systems, resilience ranks changed the most for the middle-ranked sites. In general, the wider the range of weights, the larger the changes in weighting ranks from unweighted to weighted (Table 1). For example, Fig 4 shows the relationship between the unweighted ranks and three weighted ranks. The greatest level of dispersion around the 1:1 line is for weighting system 3, in which weights ranged from 1 to 8.

Because resilience quartiles may sometimes be more useful than resilience ranks for management purposes, Table 5 shows how many sites changed resilience quartiles under each weighting system. Except for the extreme weighting system 3, over 80% of sites stayed in the same resilience quartile in the other ecologically based scenarios (systems 4–9), and in no other system did sites change by more than one quartile. Unsurprisingly, the system with the greatest number of quartile changes was the one with the widest range of weights (system 3).

**Table 5:** Percent of sites that changed or maintained their resilience quartiles under nine indicator weighting systems in comparison with unweighted indicators (n = 103). For descriptions of each weighting system, refer to Table 1. Quartile 1 is the quarter of sites with the highest resilience; quartile 4 is the quarter of sites with the lowest resilience.

Quartile change	Weighting 1	Weighting 2	Weighting 3	Weighting 4	Weighting 5	Weighting 6	Weighting 7	Weighting 8	Weighting 9
**1 to 1**	23.3%	21.4%	17.5%	23.3%	22.3%	24.3%	23.3%	23.3%	22.3%
**1 to 2**	1.9	3.9	4.9	1.9	2.9	1.0	1.9	1.9	2.9
**1 to 3**	0.0	0.0	2.9	0.0	0.0	0.0	0.0	0.0	0.0
**2 to 1**	1.9	2.9	6.8	1.9	2.9	1.0	1.9	1.9	2.9
**2 to 2**	23.3	14.6	13.6	21.4	20.4	21.4	19.4	20.4	17.5
**2 to 3**	0.0	7.8	3.9	1.9	1.9	2.9	3.9	2.9	4.9
**2 to 4**	0.0	0.0	1.0	0.0	0.0	0.0	0.0	0.0	0.0
**3 to 1**	0.0	1.0	1.0	0.0	0.0	0.0	0.0	0.0	0.0
**3 to 2**	0.0	6.8	6.8	1.9	1.9	2.9	3.9	2.9	4.9
**3 to 3**	24.3	12.6	13.6	21.4	20.4	20.4	19.4	20.4	18.4
**3 to 4**	0.0	3.9	2.9	1.0	1.9	1.0	1.0	1.0	1.0
**4 to 2**	0.0	0.0	0.0	0.0	0.0	0.0	0.0	0.0	0.0
**4 to 3**	0.0	3.9	3.9	1.0	1.9	1.0	1.0	1.0	1.0
**4 to 4**	25.2	21.4	21.4	24.3	23.3	24.3	24.3	24.3	24.3
**Sites with unchanged quartile**	96.1%	69.9%	66.0%	90.3%	86.4%	90.3%	86.4%	88.3%	82.5%

### Stressors

**Fig 5:**
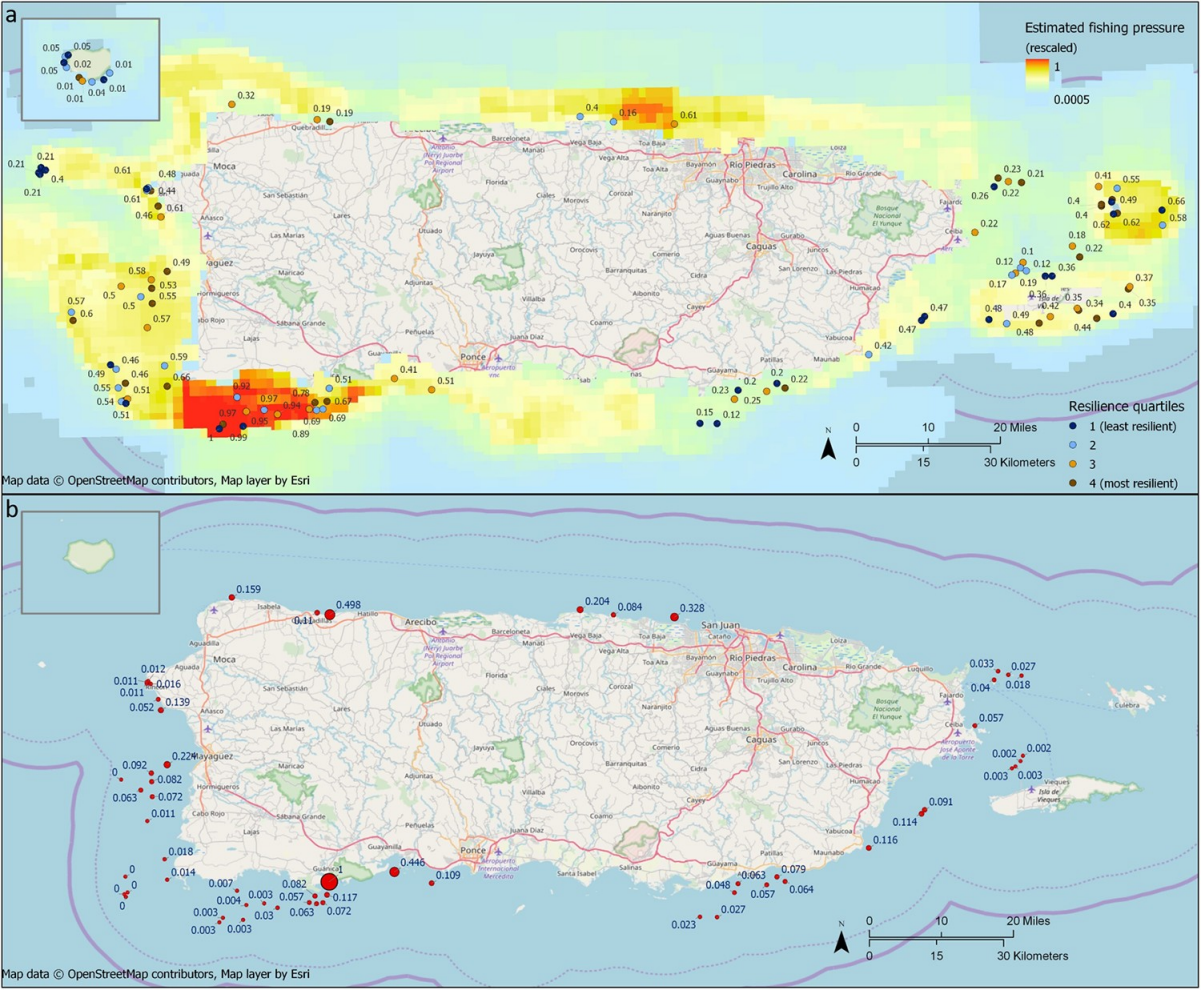
Fishing and land-based sources of pollution (LBSP) stressors, rescaled to a maximum of 1. a) Estimated rescaled potential fishing pressure from lines, nets, traps, and diving (Shivlani & Koeneke, 2011). Numbers are relative fishing pressure. Reef survey sites (n = 103) are coded by resilience quartile. Isla de Mona is the inset in the top left. Resilience quartiles are based on resilience calculated without the temperature variation indicator. b) Rescaled LBSP exposure (sediment and nitrogen) at main island survey sites within 15 km of a river mouth (n = 59). Values are rescaled LBSP (maximum value = 1).

There was a nearly 20,000-fold difference in fishing pressure across survey sites (Fig 5a). The highest-pressure area was in southwest Puerto Rio, followed by the north-central coast. Similarly, there was a 22,000-fold difference in the average land-based pollution scores across sites (Fig 5b). The distribution of land-based pollution exposure was extremely skewed; 53 of the 59 sites with scores were less than 0.2. Thus, this stressor was dominated by the few sites that were close to large rivers, due to the combined use of site distance from river mouth and pollutant load (generally greater in larger watersheds) in the calculation of LBSP exposure. Our exclusion of the contributions of coastal development and sewage outfalls could result in mis-ranking some reef sites.

### Management queries

**Fig 6:**
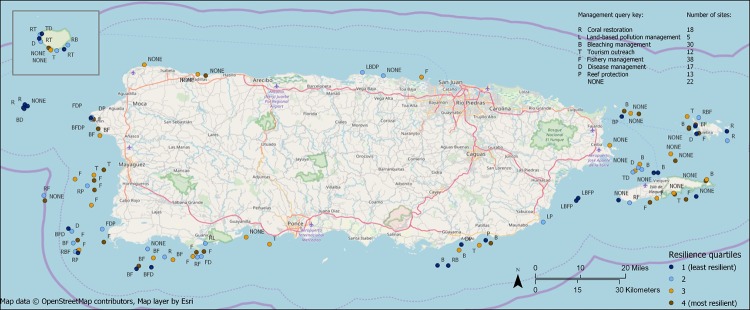
Reef survey sites (n = 103) showing which management queries applied to each site. Refer to Table 2 for criteria used in each query. Isla de Mona is the inset in the top left. Resilience quartiles are based on resilience calculated without the temperature variation indicator.

Twenty two of the 103 sites did not have any applicable management actions, 43 sites fit one action, 28 sites fit two actions, six sites fit three actions, four sites fit four actions, and no sites fit more than four actions (Fig 6). The most common action was fishery management (38 sites) and the least common was management of land-based sources of pollution (5 sites). One reason that the LBSP management criteria fit fewer sites was that only the 59 sites around the main island had LBSP estimates. Had we been able to generate LBSP estimates for the outlying islands, presumably some of those sites would have fit the LBSP management criteria, too.

### Bleaching exposure and vulnerability

**Fig 7:**
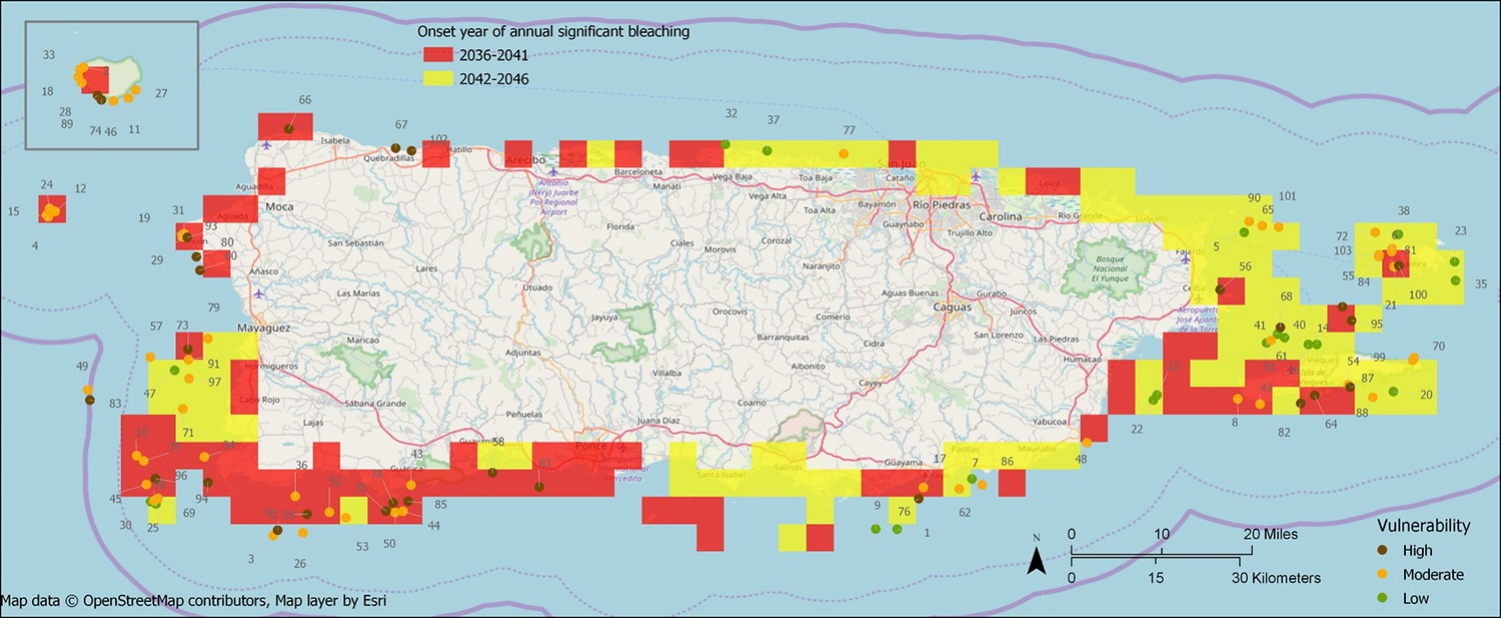
Reef survey sites (n = 103) with resilience rank (without temperature variation indicator), year of onset for annual significant bleaching (ASB) under RCP8.5 scenario, and site vulnerability category. Isla de Mona is the inset in the top left. ASB onset estimates are from van Hooidonk et al. (2016). Low exposure sites have ASB onset in 2042 or later while high exposure sites have ASB onset in 2041 or earlier. Vulnerability matrix: combination of exposure (year of onset of ASB) and resilience to produce relative vulnerability categories: high, moderate, and low. High resilience sites are sites in the two most resilient quartiles while low resilience sites are in the two least resilient quartiles.

Estimates of the year of onset of ASB for the survey sites using [34] ranged from 2036 to 2046, with the most common year being 2039 (41 sites). There were 20 low vulnerability sites, 55 moderate vulnerability sites, and 28 high vulnerability sites. Sites were intermixed across Puerto Rico by the three levels of relative vulnerability (Fig 7).

All data—including NCRMP site information provided by NOAA, raw and rescaled indicators, resilience scores, relative stressor values, management queries by site, and vulnerability assignments, for all indicator weighting systems—are included in S2 Table.

## Discussion

We assessed the relative resilience of reefs to warming ocean temperatures at 103 sites around Puerto Rico. Because Puerto Rico has not had a survey specifically designed to measure resilience, we used this as an opportunity to explore whether the same kinds of analyses could be performed with a desktop assessment as during an assessment with a field component. We were able to perform many of the same analyses undertaken in recent assessments of other U.S. coral reef jurisdictions because the reef survey we used (National Coral Reef Monitoring Program (NCRMP)) included data for the majority of necessary resilience indicators. The two exceptions were recruitment and connectivity, which we could not explore because NCRMP did not collect the necessary information on coral recruits and high-resolution near-shore current data were not available. However, we were able to perform two analyses that previous assessments had not conducted because of the probabilistic design of the survey we used: interpolation of a resilience surface from resilience survey points, and evaluation of the level of spatial aggregation of sites by resilience quartile. While we have demonstrated that it is possible to conduct a complete resilience assessment using a survey that was not designed for that purpose, such assessments will be heavily affected by the indicators that can be created from the source survey and its spatial coverage and resolution. In Puerto Rico, spatial coverage was fairly complete.

For this assessment, we mapped resilience in two ways: as point estimates at survey sites, and as a resilience surface that interpolated resilience around Puerto Rico using the sites’ resilience scores. In conjunction with statistical testing of resilience aggregation by quartile, these maps suggest a high level of intermixing of different resilience levels at sites in close proximity. Thus, no one region of Puerto Rico was clearly the most or least resilient, except perhaps for Desecheo National Wildlife Refuge, at which all four sites were in the highest resilience quartile. Although the actual resilience scores and relative scores of sites depend on how well the NCRMP survey captures indicators’ variation across reefs, this finding of high spatial variability in resilience is probably robust to that because this appears to be an island-wide property of the data. Where reefs of vastly different resilience are close to each other, they will likely have different management objectives and needs. Ultimately, this requires fine-scale reef management.

To support resilience-based management of Puerto Rico’s coral reefs, we demonstrated how a manager can query indicators, resilience scores, and stressors to identify sites at which certain adaptation actions would be appropriate, akin to [9]. Some actions might be designed to restore lost resilience (e.g., coral restoration to regenerate reef structure and function), others might be designed to protect existing resilience (e.g., land-based pollution management, tourism outreach, fishery management, reef protection), and others might be designed to improve resilience by boosting recovery potential (e.g., bleaching and disease management. If an action decreases sensitivity or increases adaptive capacity (or both), this supports resilience.

While the management queries presented here capture reef conditions as of the most recent data collection and use the best available information, they can also be modified in a few ways as new information becomes available. First, new queries can be added, or existing ones removed, based on managers’ preferences; the analysis (including query) R code and data are available in the supplementary materials. Several of the queries focus on reducing non-climate stressors (e.g., LBSP management, fishing management), but this is just one of at least seven general categories of adaptation strategies recommended in the literature [35]. Second, the criteria for the queries could be changed. For example, the disease management query could include a criterion about coral diversity. In any case, this management query technique is flexible, giving managers additional latitude in how to construct their actions.

Another elaboration on the management action queries might be to use different queries or criteria in different parts of Puerto Rico, based on local conditions or objectives. Using the same data, the assessment could be conducted at scales at which local management occurs. While our Puerto Rico-wide assessment is relevant for territory-wide planning and high-level scanning for options, a localized assessment would support management at smaller scales. For example, at the Puerto Rico-wide scale, of the five sites that met the LBSP management query criteria, three are off southeast Puerto Rico (the only sites in the area), suggesting that those might be good places to initially focus on LBSP management if a new project or focal watershed is being considered. Similarly, a few clusters of sites met the restoration target criteria in southwest Puerto Rico and off Culebra; those might be natural areas for conducting local assessments for starting coral restoration projects. Although this assessment can suggest some places to prioritize looking, whether these areas are truly conducive to the suggested management actions depends on many other factors.

Although summarizing sites by aggregate resilience score is useful, it entails some problems. First, it omits the information contained by the individual indicators, and second, it introduces additional assumptions to the analysis. Regarding the first issue, sites can achieve very high resilience by having high scores for every indicator or by having higher scores for more indicators than do other sites. For this assessment, the most resilient sites did not have the highest resilience scores for every indicator. For example, three of the indicators at the most resilient site had the highest scores while the other four indicators had moderate or low values relative to other sites. This was true for other very high resilience sites as well. It suggests that no specific site was “most resilient” in every aspect; even the most resilient sites could increase aspects of their resilience to levels found at other sites. This has been the case in previous assessments, for example, in West Hawai’i [14] and in the Florida Keys [15]. In both of those assessments, the most resilient sites were very low for one or more indicators. More generally, resilience scores may not be correlated with measures like bleaching response while individual indicators are, thereby masking important associations for which the resilience indicators were selected in the first place. For example, [21] found that responses to bleaching were correlated with particular resilience indicators (such as community thermal tolerance and thermal history), but not correlated with the composite resilience score.

Regarding the second issue—summarizing sites by resilience score introducing additional assumptions to the analysis—we undertook the weighting sensitivity analysis because of the uncertainty associated with combining indicators into a composite score. This uncertainty arises from the lack of a formal model for combining indicators in the region that accounts for non-linearities and context-dependence [3]. In effect, by using a variety of weights, we contrasted several non-mechanistic ecological models against our null model (unweighted indicators). The indicator weighting sensitivity analysis suggested that indicator weighting within the range of weights suggested by [12] will affect the ranks of the mid-resilience sites more than the ranks of extreme sites (Fig 4). Nevertheless, the results of the assessment were generally robust to ecologically based weighting systems, especially if resilience quartiles were used rather than ranks. The different weighting systems appeared to have little effect on the more derived products of the resilience assessment, such as the management queries or vulnerability analysis. For example, the distribution of sites among vulnerability levels was very similar using the nine weighting systems, although the sites in each category differed somewhat depending on the weighting system (S2 Table). In terms of management uses of the resilience assessment, using resilience quartiles for management decisions will be less affected by weighting assumptions than pure resilience ranks or scores will be. This is beneficial for managers because it means that the management queries are more robust to weighting uncertainties than are actions based directly on resilience scores or ranks.

As with assessments in other U.S. jurisdictions, having four of the seven indicators be coral-related inherently weights resilience scores towards the status of the coral community over other community or ecosystem dimensions, such as fish. The panel discussed this during indicator selection and felt it reflected what contributes to Puerto Rico’s reef resilience. Two analyses also confirmed the predominance of the status of the coral community in resilience scores. In the exploratory factor analysis, the first factor was essentially a composite of the coral indicators. Meanwhile, despite having just one indicator, herbivorous fish dominated the second factor, which explained almost as much variance as did the first factor (17.4% for the first factor vs. 14.8% for the second factor (Table 4)). Spearman correlations between indicators (Table 3) further show the lack of independence and potential redundancy between certain indicators, such as the coral and coral-associated (i.e., rugosity) indicators. While this emphasis on coral community status is standard in resilience assessments, it is necessary to be cognizant of it.

Perhaps the most important indicators that were not available for this assessment were coral recruitment and reef connectivity [5,8] and reef calcification or accretion rates [5,26]. Although connectivity was given a lower importance score by [12] than six of the indicators we used, it was still deemed important by our expert panel and has been included in resilience assessments for which there have been available data. For example, [8] used available fine-scale ocean current models and a coral recruitment indicator to identify sources and sinks for coral larvae around the Commonwealth of the Northern Mariana Islands, and [4] used coarser information to speculate on connectivity. Including connectivity allows inference about which sites are sources and sinks for larvae and enables management queries about ensuring connectivity, one of the seven general adaptation strategies of [35]. By not including process indicators like connectivity and accretion rate, assessments miss the drivers of the observed system state, capturing only the results of unmeasured drivers [3,6]. Although this assessment only includes state indicators because of the design of the available survey, it still covers a wide range of important state indicators.

Inherent in the use of resilience indicators is the assumption that reefs possessing higher scores for these indicators are more resilient to a specific type of disturbance, such as increased sea surface temperatures from climate change. [6] recommended that resilience assessments be repeated following bleaching events or large storms to test whether this is true. There has generally been little testing for individual indicators (i.e., few before- and after-disturbance studies, although see [21]). In the intervening years since the data used in this paper were collected, there has been a global bleaching event and devastating Hurricane Maria in 2017. Although there have been reef surveys since the survey this assessment is based on, they have not specifically revisited sites included in this assessment and thus cannot validate these resilience indicators for Puerto Rico. Without some sense of which of these indicators accurately indicate resilience to increasing SST in Puerto Rico, they remain largely untested hypotheses. Nevertheless, since there are no “validated” resilience indicators for Puerto Rico and many other jurisdictions, using the ones with the greatest support is the best path forward for those trying to protect and restore reefs.

Continuing to improve and verify resilience concepts will be essential to the effective pursuit of ‘climate-smart’ management [36]. Within the context of the climate-smart adaptation planning cycle for coral reefs [35,37], this work falls under step 2, assessing climate impacts and vulnerabilities. Both resilience and vulnerability assessments provide critical information to support identifying adaptation options (step 4) and evaluating and selecting priority adaptation actions (step 5), in both cases as an information input into processes such as the Adaptation Design Tool [37]. For resilience assessments to be useful for those steps, they need to be at the correct spatial scale and resolution. Although this assessment can help with broader scale prioritization and goal setting, it is probably at a larger scale than is ideal for local actions because of its comparison of all sites across Puerto Rico. One way to facilitate making this assessment useful at scales smaller than Puerto Rico would be to create an online tool that allows users to select a subset of monitoring sites or geographic areas and perform the assessment for their custom-selected area or pool of sites.

## Supporting information

S1 Table List of resilience indicators considered by expert panel for inclusion in resilience assessment. All were indicators that could be calculated from the available National Coral Reef Monitoring Program 2014 survey.
(DOCX)

S2 Table Raw and rescaled indicators, resilience scores, ranks and quartiles, stressor values, management queries, onset year of annual significant bleaching, vulnerability category, and NCRMP site info for each site for each indicator weighting system. (XLSX)

S3 Table Data sources for OpenNSPECT model. (DOCX)

S4 Table Correlation between OpenNSPECT flow and sediment output and USGS gage data (r^2^). The default comparison is without dammed sites and without hurricane years.
(DOCX)

S1 Text Parameters used for geospatial analyses. (DOCX)

S2 Text Methods for calculating land-based sources of pollution around Puerto Rico. (DOCX)

S1 Fig Relationship between OpenNSPECT output and USGS stream gage data. Results use all USGS gages (including ones near dams) and all years of data (including hurricane years). a) Stream flow. b) Sediment.
(TIF)

S2 Fig Flow, sediment, and nitrogen at river and stream mouths, as output from OpenNSPECT. Model endpoints for all rivers and streams with more than 183 kg N/day were aligned with National Hydrography Dataset (NHD) flowlines and combined to a single point when needed. Outputs from OpenNSPECT are meant to be used relative to each other; the display of actual output values is merely illustrative. a) Flow (mean annual discharge in liters). b) Sediment (mean annual load in kg). c) Nitrogen (mean annual load in kg).
(TIF)

S1 Information File R script for conducting resilience assessment. (RMD)
