# Supplementary material for: Resilience assessment of Puerto Rico’s coral reefs to inform reef management
Source: PLoS One. 2019 Nov 5;14(11):e0224360. doi: 10.1371/journal.pone.0224360 (PMC6830742; doi:10.1371/journal.pone.0224360)
Supplement: S1 Table — All were indicators that could be calculated from the available National Coral Reef Monitoring Program 2014 survey. (DOCX) [file pone.0224360.s001.docx]

| Potential indicator number | Indicator name | Category | Used in previous resilience assessments | Used in BCG report | Pre-mtg. tier | Number of stations available |
| --- | --- | --- | --- | --- | --- | --- |
| 1 | Temperature variation | Other | ✓ | ✓ | Previous | 230 |
| 2 | Fleshy macroalgae percent cover | Algae | ✓ | ✓ | Previous | 230 |
| 3 | Coral percent cover (total) | Coral | ✓ | ✓ | Previous | 230 |
| 4 | Percent bleaching-resistant coral species | Coral | ✓ | ✓ | Previous | 103 |
| 5 | Coral diversity (Simpson index) | Coral | ✓ | ✓ | Previous | 103 |
| 6 | Coral disease prevalence | Coral | ✓ | ✓ | Previous | 103 |
| 7 | Average herbivore biomass across functional groups | Herbivores | ✓ | ✓ | Previous | 230 |
| 8 | Coral population structure (colony size) | Coral |  | ✓ | 1 | 103 |
| 9 | Reef depth | Other |  | ✓ | 1 | 230 |
| 10 | Reef rugosity/topographic complexity | Other | Used in later assessments | ✓ | 1 | 230 |
| 11 | Percentage of live coral tissue on colonies | Coral |  | ✓ | 2 | 103 |
| 12 | *Diadema* *antillarum* presence/absence or abundance | Herbivores |  | ✓ | 2 | 230 |
| 13 | Parrotfish taxa richness | Herbivores |  | ✓ | 2 | 230 |
| 14 | Fish taxa richness | Other fish |  | ✓ | 2 | 230 |
| 15 | Fish total biomass | Other fish |  | ✓ | 2 | 230 |
| 16 | Within-family fish diversity | Other fish |  | ✓ | 2 | 230 |
| 17 | Piscivorous fish presence/absence | Other fish |  | ✓ | 2 | 230 |
| 18 | Damselfish density/percent of fish community | Other fish |  | ✓ | 2 | 230 |
| 19 | Coral percent cover (by species) | Coral |  | ✓ | 3 | 230 |
| 20 | Acropora thicket and boulder coral presence | Coral |  | ✓ | 3 | 230 |
| 21 | Distance from shore | Other |  | ✓ | 3 | 230 |
| 22 | Distance from shelf | Other |  | ✓ | 3 | 230 |
| 23 | Gorgonians present but sub-dominant | Other |  | ✓ | 3 | 230 |
