# Supplementary material for: Resilience assessment of Puerto Rico’s coral reefs to inform reef management
Source: PLoS One. 2019 Nov 5;14(11):e0224360. doi: 10.1371/journal.pone.0224360 (PMC6830742; doi:10.1371/journal.pone.0224360)
Supplement: S3 Table — (DOCX) [file pone.0224360.s003.docx]

**S3 Table- Data sources for OpenNSPECT model**

| **Model input** | **Data type** | **Source** |
| --- | --- | --- |
| HUC10 boundary | Shapefile | NHDplus V2: <http://www.horizon-systems.com/NHDPlus/NHDPlusV2_21.php> |
| Land use | Raster | Coastal Change Analysis Program (C-CAP) 2010: <https://coast.noaa.gov/digitalcoast/tools/lca> |
| Elevation (DEM) | Raster | Six 1/3 arc second tiles merged: <http://viewer.nationalmap.gov/basic/> |
| Annual precipitation | Raster | From Greg Morris Engineering, Figures 5 and 6 in 2009 report “Minimum instream flow estimation at ungaged stream sites in Puerto Rico” |
| R-factor | Raster | OpenNSPECT website: <https://coast.noaa.gov/data/digitalcoast/zip/R-Factor-PR.zip> |
| Hydrologic soil group (HSG) and K-factors | Shapefile | Seven SSURGO files, processed as directed in the OpenNSPECT guide and merged into an island-wide shapefile. To be conservative, soil classes missing HSGs were defaulted to group D and soil classes missing K-factors were defaulted to 0.3. <http://websoilsurvey.sc.egov.usda.gov/App/WebSoilSurvey.aspx> |
| Raining days | Value for each HUC10 | Calculated according to <https://geozoneblog.wordpress.com/2014/04/22/raining-days/> |
