# Supplementary material for: Resilience assessment of Puerto Rico’s coral reefs to inform reef management
Source: PLoS One. 2019 Nov 5;14(11):e0224360. doi: 10.1371/journal.pone.0224360 (PMC6830742; doi:10.1371/journal.pone.0224360)
Supplement: S4 Table — The default comparison is without dammed sites and without hurricane years. (DOCX) [file pone.0224360.s004.docx]

**S4 Table- Correlation between OpenNSPECT flow and sediment output and USGS gage data (r^2^).** The default comparison is without dammed sites and without hurricane years.

| Without dammed sites | Without hurricane years | Flow | Sediment |
| --- | --- | --- | --- |
| No | No | 0.66 | 0.37 |
| No | Yes | 0.52 | 0.25 |
| Yes | No | 0.75 | 0.26 |
| Yes | Yes | 0.75 | 0.31 |
