# Supplementary material for: Resilience assessment of Puerto Rico’s coral reefs to inform reef management
Source: PLoS One. 2019 Nov 5;14(11):e0224360. doi: 10.1371/journal.pone.0224360 (PMC6830742; doi:10.1371/journal.pone.0224360)
Supplement: S1 Text — (DOCX) [file pone.0224360.s005.docx]

**S1 Text:**

**Analysis parameters:**

For the inverse distance weighting raster interpolation tool in ArcGIS Pro v2.0, we used the following parameters: 0.02 x 0.02 degrees (approximately 2.2 x 2.2 km) pixels, power=2, search radius=variable, number of points=5).

Spearman rank-correlation coefficients between all indicators were calculated using chart.Correlations function in the PerformanceAnalytics package of R.

Exploratory factor analysis (EFA) was conducted using the factanal function in the stats package to identify latent variables in the resilience assessment. We retained factors with an eigenvalue > 1 using a scree plot (scree.plot function in psy package) for latent variable interpretation.
