# Supplementary material for: Resilience assessment of Puerto Rico’s coral reefs to inform reef management
Source: PLoS One. 2019 Nov 5;14(11):e0224360. doi: 10.1371/journal.pone.0224360 (PMC6830742; doi:10.1371/journal.pone.0224360)
Supplement: S2 Text — (DOCX) [file pone.0224360.s006.docx]

**S2 Text:**

**Land-based sources of pollution modeling methods:**

There were two main stages to estimating the delivery of land-based sources of pollution (LBSP) to reefs around Puerto Rico. First, we created watershed models for flow, sediment load, and nitrogen load to estimate their output at river and stream mouths (collectively called river mouths or pour points henceforth) around Puerto Rico. We validated the modeled flows and sediment loads using US Geological Survey (USGS) river gage data. Second, we estimated exposure of survey sites to sediment and nitrogen using a simple dispersion model.

*Watershed model*

We used OpenNSPECT [31] to estimate flow, sediment load, and nitrogen load at river mouths around Puerto Rico. OpenNSPECT is a free, GIS-based, simplified watershed model that estimates in-stream flow and pollutant loads, including sediment, nitrogen, phosphorus, and metals. It is an add-in to the free GIS program MapWindow version 4.8. Although it estimates absolute values for flow and pollutants using the revised universal soil loss equation (RUSLE) and wash-off coefficients, its estimates should be interpreted as relative differences rather than as absolute values. Also, OpenNSPECT does not produce a time-series for flows and pollutants. Rather, it produces an estimate of the total flow and pollutant loads that pass each pixel during the course of a year of “average” precipitation or as a result of a single, large storm. We used OpenNSPECT for the former purpose.

We created and ran a separate OpenNSPECT model for every 21 10-digit hydrologic unit code watershed (HUC10) on Puerto Rico’s main island. We used this level of division after experimenting with using OpenNSPECT on watersheds of different sizes. We did not run OpenNSPECT on Vieques or Culebra islands because they did not have all the necessary data available. As the basis for the models, we used the NHDPlusV2 HUC10 boundary shapefile for Puerto Rico. We used ArcMap to create a separate shapefile for each HUC10 boundary, buffered to 250 m per the recommendations of the OpenNSPECT manual.

We followed the OpenNSPECT data acquisition and preparation manual to pre-process our model inputs (sources shown in S3 Table) [32]. We projected every input file to UTM Zone 20N, then used the “Assign Projection to Grids” tool in OpenNSPECT to align all input rasters to the same grid. Next, we used the “Clip and Project New Data” tool on the elevation, land use, precipitation, and R-factor input files for each HUC10 model, with a 30 m cell size and a buffer size of 50 cells. After that, we used the OpenNSPECT “Watershed Delineations” tool (“Subwatershed size” set to “small”) to obtain stream channels for each HUC10. The stream channels produced by OpenNSPECT generally matched National Hydrography Dataset (NHD) flowlines, except in low-slope, hydrologically altered lowlands. Finally, we clipped the SSURGO shapefile to each buffered HUC10 shapefile to get the soil input (hydrologic soil group and K-factor) for the models.

**S3 Table- Data sources for OpenNSPECT model**

| **Model input** | **Data type** | **Source** |
| --- | --- | --- |
| HUC10 boundary | Shapefile | NHDplus V2: <http://www.horizon-systems.com/NHDPlus/NHDPlusV2_21.php> |
| Land use | Raster | Coastal Change Analysis Program (C-CAP) 2010: <https://coast.noaa.gov/digitalcoast/tools/lca> |
| Elevation (DEM) | Raster | Six 1/3 arc second tiles merged: <http://viewer.nationalmap.gov/basic/> |
| Annual precipitation | Raster | From Greg Morris Engineering, Figures 5 and 6 in 2009 report “Minimum instream flow estimation at ungaged stream sites in Puerto Rico” |
| R-factor | Raster | OpenNSPECT website: <https://coast.noaa.gov/data/digitalcoast/zip/R-Factor-PR.zip> |
| Hydrologic soil group (HSG) and K-factors | Shapefile | Seven SSURGO files, processed as directed in the OpenNSPECT guide and merged into an island-wide shapefile. To be conservative, soil classes missing HSGs were defaulted to group D and soil classes missing K-factors were defaulted to 0.3. <http://websoilsurvey.sc.egov.usda.gov/App/WebSoilSurvey.aspx> |
| Raining days | Value for each HUC10 | Calculated according to <https://geozoneblog.wordpress.com/2014/04/22/raining-days/> |

The final pre-processing tool for OpenNSPECT was the “Precipitation Scenarios” tool. In addition to the clipped precipitation raster, OpenNSPECT needed “raining days” values, which is the number of days each year with sufficient rain to cause runoff. Each HUC10 was assigned one value for “raining days”, which was calculated according to <https://geozoneblog.wordpress.com/2014/04/22/raining-days/> using 1981-2010 monthly normal precipitation data from NOAA’s National Center for Environmental Information (NCEI) rain gages (<http://www.ncdc.noaa.gov/cdo-web/datasets>), the dominant hydrologic soil group (HSG) in the HUC10, and the land use composition in the HUC10. For the two HUC10s that did not have any NCEI data (2101000303 and 2101000503), we used the average raining days from the nearest stations in the adjacent HUC10s. This resulted in each model having its own “raining days” input value. Of course, not everywhere in a HUC10 actually has the same number of “raining days” but this was a necessary simplification for the model. For the “Precipitation Scenarios” tool, “Time period” was “Annual” and “Type” was “Type III”.

For each HUC10 model, we loaded the appropriate pre-processed files, selected the “Include local effects” output box, selected nitrogen as a pollutant to model, and turned on the erosion module. This produced rasters of flow, nitrogen and sediment loads (kg/day), and nitrogen and sediment concentrations (mg/L) at every pixel in every HUC10. Using the “Mosaic to new raster” tool in ArcMap, the HUC10 outputs for flow and each pollutant load were combined into island-wide rasters. We did not use OpenNSPECT’s total suspended sediment (TSS) output because it is less accurate than the sediment output (D. Eslinger, pers. comm). These rasters constituted the output of the watershed model.

To validate the OpenNSPECT watershed model, we compared modeled flow and sediment values to USGS National Water Information System (NWIS) measurements. The goal of this validation was to make sure that there was a linear relationship between modeled and observed values (because OpenNSPECT is most useful for relative outputs), not that the modeled and observed values actually matched each other. We did not validate OpenNSPECT’s nitrogen output because none of the USGS gages had sufficient nitrogen records and other consistent, island-wide measurements were not available.

To perform this validation, we downloaded annual flow averages from every USGS gage that had more than 10 years of data and sediment load data from every gage that had more than five years of data. We set these requirements for number of years because they balanced the need to capture inter-annual variability with the need to have an adequate number and coverage of sites across the island for validation. We used a lower year threshold for sediment than for flow because fewer stations had sediment data to begin with and requiring 10 years would have very severely restricted our validation dataset. These thresholds led to 96 flow validation stations and 40 sediment validation stations. These validation sites were scattered throughout Puerto Rico and are not generally at the coast, where validation of OpenNSPECT would be most beneficial. We averaged the annual flow and sediment loads for each USGS gage that had data and compared those values to the values produced by OpenNSPECT at the same location.

Because OpenNSEPCT does not include the effects of dams on flow or pollutants but USGS measurements do, comparison of USGS gage data to OpenNSPECT output can be thrown off by including gages heavily influenced by dams and reservoirs. Thus, we also examined the relationship between modeled and observed flow and sediment loads without five gages near dams: 50027750, 50045010, 50047560, 50059050, and 50148890.

Finally, because we used OpenNSPECT to model flow and pollutant loads from a year of average precipitation, its output does not include the effects of extreme events. Thus, to further make the USGS gage data more comparable to the OpenNSPECT output, we removed the annual averages from the gages during which there were hurricanes in Puerto Rico (1956, 1975, 1979, 1984, 1989, 1993, 1995, 1996, 1998, 2001, and 2004). We then re-examined the relationship between OpenNSPECT’s output and USGS sites not at dams and without those years of data. The r^2^ statistics under these various conditions are in S4Table. In general, excluding the dams improved the fit of OpenNSPECT for flow but excluding dammed sites and hurricane years worsened the fit for sediment. Beyond the r^2^, OpenNSPECT’s flow output was well within the same order of magnitude as the USGS values while sediment estimates were about 100x greater than USGS values (S1 Fig). This two-orders-of-magnitude discrepancy did not matter for the next phase of the model, however, because we used the relative sediment loads, not the absolute values.

**S4 Table- Correlation between OpenNSPECT flow and sediment output and USGS gage data (r^2^).** The default comparison is without dammed sites and without hurricane years.

| Without dammed sites | Without hurricane years | Flow | Sediment |
| --- | --- | --- | --- |
| No | No | 0.66 | 0.37 |
| No | Yes | 0.52 | 0.25 |
| Yes | No | 0.75 | 0.26 |
| Yes | Yes | 0.75 | 0.31 |

**Supplementary Fig 1. Relationship between OpenNSPECT output and USGS stream gage data.** Results use all USGS gages (including ones near dams) and all years of data (including hurricane years). a) Stream flow. b) Sediment.

The final step before modeling coastal dispersion of sediment and nitrogen was to determine flow and sediment and nitrogen loads at river and stream mouths around Puerto Rico. To do this, we identified every river coastal pour point with more than 183 kg of nitrogen according to OpenNSPECT (0.5 kg/day). Because of imperfections in OpenNSPECT’s delineations (especially in low-slope coastal areas), not all of these locations corresponded to real river or stream mouths. Thus, we tried to locate where every OpenNSPECT pour point actually released at the coast using NHD flowlines and aerial imagery. Some river and stream mouths corresponded to multiple OpenNSPECT pour points; we aggregated these by river mouth in ArcMap. Some river mouths were consolidated if they drained to the same coastal embayment with a narrow opening, in which case the combined river mouth was put at the midpoint of the individual river mouths. Ultimately, this produced 121 river and stream mouths, each of which had their own flow, nitrogen load, and sediment load (S2 Fig).

**Supplementary Fig 2. Flow, sediment, and nitrogen at river and stream mouths, as output from OpenNSPECT.** Model endpoints for all rivers and streams with more than 183 kg N/day were aligned with National Hydrography Dataset (NHD) flowlines and combined to a single point when needed. Outputs from OpenNSPECT are meant to be used relative to each other; the display of actual output values is merely illustrative. a) Flow (mean annual discharge in liters). b) Sediment (mean annual load in kg). c) Nitrogen (mean annual load in kg).

*Dispersion model*

We used a basic model to estimate dispersion of LBSP to reef surveys sites around Puerto Rico. We limited dispersion to NCRMP sites that were within 15 km of one or more pour points, limiting the model to 59 sites (we could not create a LBSP model for Vieques and Culebra). Then we identified the seven closest pour points within 15 km to each NCRMP site. We assumed that most of the LBSP at each site would come from the seven closest river and stream mouths. Next, we rescaled the distances between pour points and reef sites, with the shortest distance across all points being 1 and longer distances being proportionately smaller (e.g., a survey site 10 km from a pour point would be rescaled to 0.1). We did this in order to weight the pour points by their proximity to survey sites. Using this approach, each pour point had one rescaled distance for each reef site that was within 15 km, i.e., each pour point-reef site combination had its own rescaled distance relative to the shortest pair’s distance.

Next, we rescaled the nitrogen and sediment loads from all the pour points, with the largest load for each again receiving a value of 1. We then multiplied the rescaled distance value for each pour point-reef site combination by the rescaled sediment value and multiplied the rescaled distance value by the rescaled nitrogen value. This produced separate nitrogen and sediment loads for each pour point-reef site combination. We separately summed the sediment and nitrogen loads from up to the seven pour points for each site and rescaled each of these 1, producing single rescaled sediment and nitrogen values for each site. To calculate a single LBSP stressor score for each site, we averaged the rescaled sediment and nitrogen scores per site and then rescaled those values.

*Model limitations and assumptions*

While the watershed model is relatively accurate (see validation discussion above), the dispersion model is unfortunately uncalibrated, largely because of the lack of systematic, standardized water quality sampling around Puerto Rico. The dispersion model has several limitations. First, it focuses on dispersion in an average year, to the exclusion of extreme events. Second, the model does not include currents; reefs in any direction from a river are equally affected by its discharge, which is generally not true around Puerto Rico. However, there is no near-shore current data at a high enough resolution available for Puerto Rico to add current information to this model. Third, the distance between river mouths and reefs is calculated as straight distances, ignoring coastal obstructions; this only matters for a subset of sites. Fourth, the results are purely relative; the model purposefully does not estimate absolute loads at sites. This is partially because of the lack of information on what sediment and nitrogen loads are biologically relevant for coral reefs and partially because, like with the resilience scores themselves, keeping LBSP data relative is useful for prioritizing management activities and identifying points of concern. Fifth, this does not include other sources of LBSP, like sewage outfalls. Unlike models that create plumes of LBSP and therefore have to select decay rates for terrestrial pollutants [37,38], this model does not assume decay rates.

Regardless of the many potential improvements to this basic model, we believe that the model is useful for LBSP and resilience management in Puerto Rico. No island-wide assessment of LBSP risk is publicly available for Puerto Rico, and this starts to fill a significant gap in managers’ knowledge. Moreover, because LBSP is a significant local stressor to reefs, it should be included in resilience assessments, and has been included in some form in many previous ones. This approach, using the best available data, provides a first approximation of that for Puerto Rico.
