# Supplementary figures and images for: Resilience assessment of Puerto Rico’s coral reefs to inform reef management
Source: PLoS One. 2019 Nov 5;14(11):e0224360. doi: 10.1371/journal.pone.0224360 (PMC6830742; doi:10.1371/journal.pone.0224360)

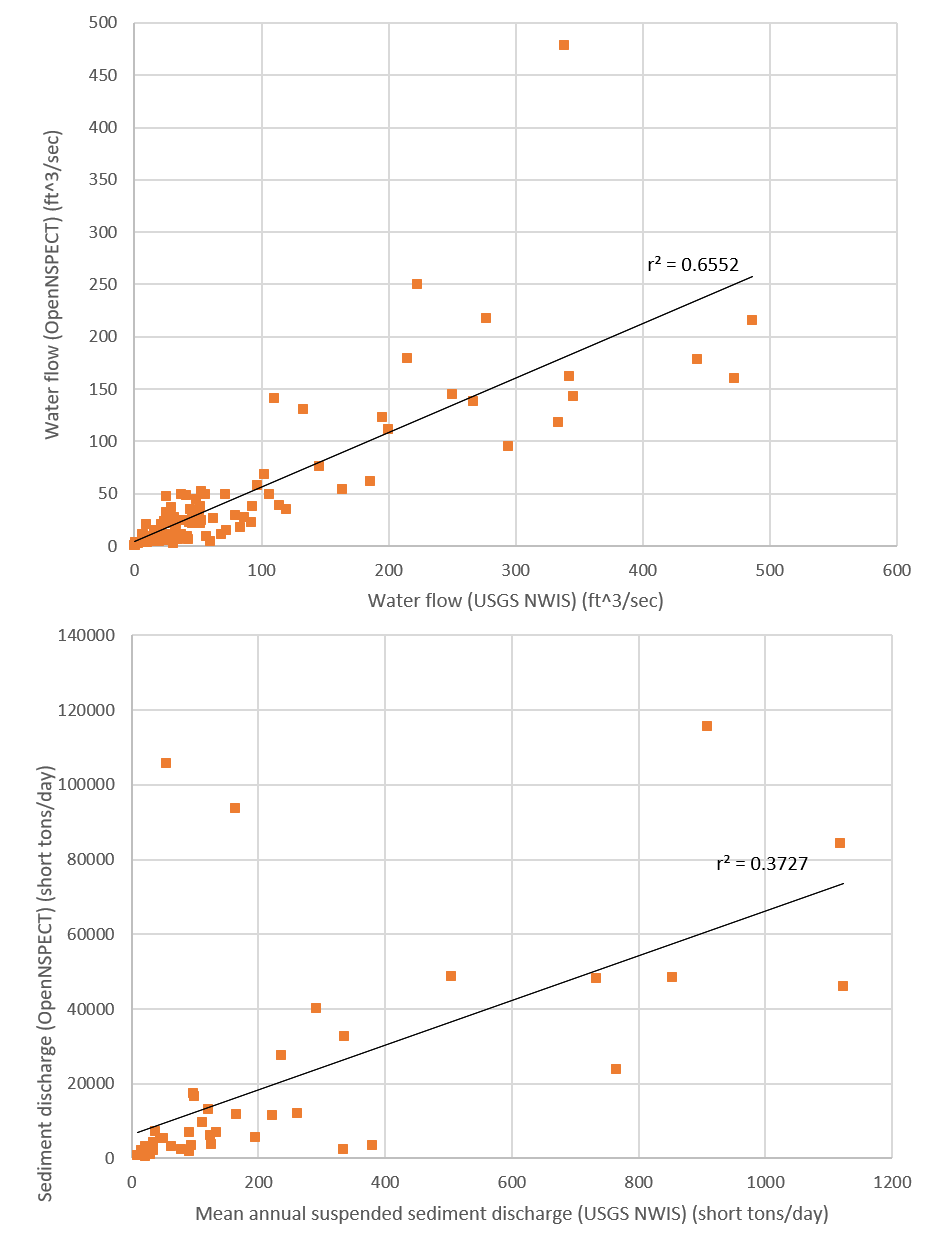

Supplement: S1 Fig — Results use all USGS gages (including ones near dams) and all years of data (including hurricane years). a) Stream flow. b) Sediment. (TIF) [file pone.0224360.s007.tif]

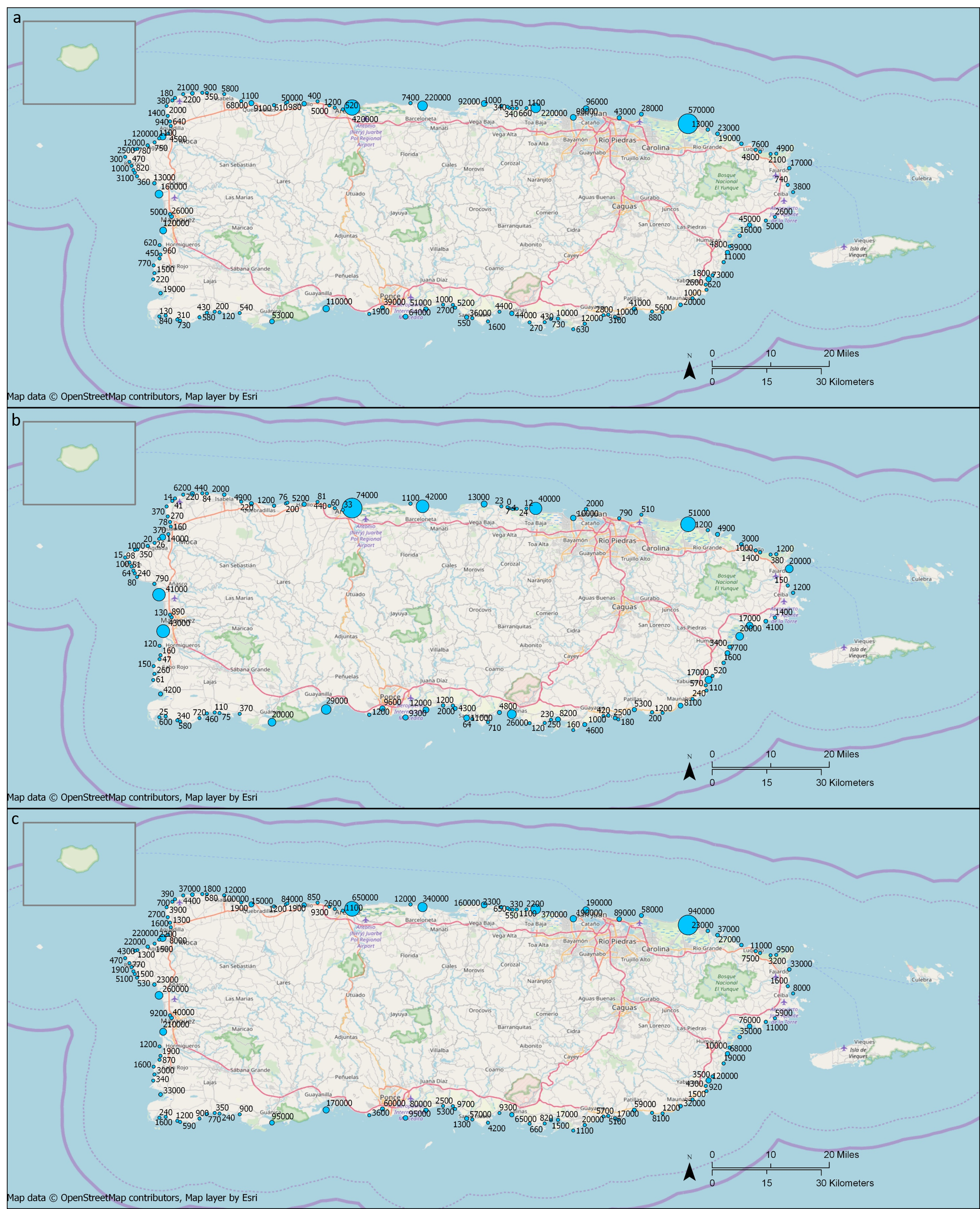

Supplement: S2 Fig — Model endpoints for all rivers and streams with more than 183 kg N/day were aligned with National Hydrography Dataset (NHD) flowlines and combined to a single point when needed. Outputs from OpenNSPECT are meant to be used relative to each other; the display of actual output values is merely illustrative. a) Flow (mean annual discharge in liters). b) Sediment (mean annual load in kg). c) Nitrogen (mean annual load in kg). (TIF) [file pone.0224360.s008.tif]
